# Roles of Wnt Target Genes in the Journey of Cancer Stem Cells

**DOI:** 10.3390/ijms18081604

**Published:** 2017-07-25

**Authors:** Jee-Heun Kim, So-Yeon Park, Youngsoo Jun, Ji-Young Kim, Jeong-Seok Nam

**Affiliations:** 1 School of Life Sciences, Gwangju Institute of Science and Technology, Gwangju 61005, Korea; jeeheun.kim@gist.ac.kr (J.-H.K.); sypark0125@gist.ac.kr (S.-Y.P.); junys@gist.ac.kr (Y.J.); 2 Cell Logistics Research Center, Gwangju Institute of Science and Technology, Gwangju 61005, Korea; 3 Silver Health Bio Research Center, Gwangju Institute of Science and Technology, Gwangju 61005, Korea; 4 Laboratory Animal Resource Center, Gwangju Institute of Science and Technology, Gwnagju 61005, Korea; jykim2533@gist.ac.kr

**Keywords:** cancer stem cell, Wnt signaling, initiation, persistence, invasion, migration, metastasis

## Abstract

The importance of Wnt/β-catenin signaling in cancer stem cells (CSCs) has been acknowledged; however, the mechanism through which it regulates the biological function of CSCs and promotes cancer progression remains elusive. Hence, to understand the intricate mechanism by which Wnt controls stemness, the specific downstream target genes of Wnt were established by analyzing the genetic signatures of multiple types of metastatic cancers based on gene set enrichment. By focusing on the molecular function of Wnt target genes, the biological roles of Wnt were interpreted in terms of CSC dynamics from initiation to metastasis. Wnt signaling participates in cancer initiation by generating CSCs from normal stem cells or non-CSCs and augmenting persistent growth at the primary region, which is resistant to anti-cancer therapy. Moreover, it assists CSCs in invading nearby tissues and in entering the blood stream, during which the negative feedback of the Wnt signaling pathway maintains CSCs in a dormant state that is suitable for survival. When CSCs arrive at distant organs, another burst of Wnt signaling induces CSCs to succeed in re-initiation and colonization. This comprehensive understanding of Wnt target genes provides a plausible explanation for how Wnt allows CSCs variation during cancer progression.

## 1. Introduction

Wnt signaling is a highly complex and evolutionarily conserved pathway that maintains pluripotency during embryonic development and regulates homeostasis in somatic stem cells from various tissues [1]. In recent decades, aberrant activation of Wnt signaling in various types of cancer has been documented and its roles in healthy tissues have been recognized. Genetic mutations that activate Wnt signaling reportedly contribute to cancer initiation [2], and nuclear accumulation of the Wnt signaling molecules β-catenin and lymphoid enhancer-binding factor 1 (LEF1) have been shown to be positively correlated with poor clinical outcomes, such as cancer progression, invasion, metastasis, and recurrence, resulting in low survival rates [2,3,4]. Accordingly, multiple studies on Wnt signaling have reported specific mechanisms that promote cancer initiation and progression and can therefore be investigated as therapeutic targets. In these studies, cancer stem cells (CSCs) have emerged as key players in Wnt-mediated carcinogenesis of various types. CSCs are a subpopulation of cancer cells with properties, such as self-renewal, slow cell cycle, persistent proliferation, homing, and mobilization, similar to those of normal stem cells and are central mediators of radio- and chemo-resistance in cancers as well as recurrence and metastasis [5,6]. Growing evidence has indicated increased Wnt signaling in CSCs compared with that in non-CSCs in multiple solid cancers and leukemia. Similarly, CSCs have elevated expression of Wnt downstream molecules compared with that in non-CSCs, as indicated by the high expression of frizzled receptors (FZD4/5) and increased sensitivity to Wnt3a-induced canonical Wnt signaling [7]. Moreover, Wnt signaling inhibition using genetic modifications or small molecule inhibitors has been shown to limit cancer stemness [8]. Specifically, deletion of the β-catenin gene results in complete regression of CD34^+^ CSCs in skin tumors. Conversely, expression of a non-degradable β-catenin expands the CSC population [9]. In the context of Wnt ligand secretion, inhibition of porcupine, which palmitoylates Wnt ligands for secretion, effectively decreases colony formation by limiting long-term self-renewal [10]. Similarly, the specific antibody OMP-18R5 blocks the binding of Wnt ligands to FZD [11] and the small molecule inhibitor CWP23228 prevents the formation of β-catenin/T-cell factor (TCF)/LEF complexes, leading to significant suppression of cancer growth, metastasis, and chemo-resistance through CSC inhibition in breast [12] and liver cancers [8]. Although the effects of Wnt on CSC stemness have been investigated in numerous studies, recent studies have suggested that Wnt signaling also plays roles in the generation of CSCs from normal stem cells and cancer cells that lack stemness. Accordingly, loss of adenomatous polyposis coli (APC) elevates the nuclear accumulation of β-catenin in leucine-rich repeat-containing G-protein-coupled receptor 5 LGR5^+^ normal stem cells and triggers neoplasia by transforming these cells into CSCs [13]. In addition, sustained high level of Wnt signaling leads to the transformation of differentiated gastrointestinal cells, which expressing high levels of doublecortin-like kinase (DCLK1), into CSCs [14]. Hence, Wnt signaling likely plays important roles in the initiation and maintenance of CSCs. However, although phenotypes and consequences of altered Wnt signaling have been reported, details of the associated regulatory mechanisms in CSCs remain unknown. Contributions of Wnt signaling to CSC initiation, persistence, resistance, invasion, and metastasis have been characterized in multiple studies, and upon CSC initiation, persistent growth in primary regions follows enhanced survival, reduced apoptosis, and altered metabolic activities in CSCs and in bulk tumor cells [2,3,4]. Subsequently, the epithelial-to-mesenchymal transition (EMT) allows entry into the process of invasion comprising detachment, intravasation, migration, and extravasation stages [2]. CSCs then progress to metastasis through dormancy, re-initiation, escape of immune surveillance, and establishment of microenvironments. In addition, defense mechanisms against chemo- and radiotherapy are highly activated in CSCs, which desensitize cells to DNA strand breaks, cell cycle arrest, and cytoskeleton or microtubule arrest, allowing continued survival and metastasis [5,6]. In this review, we investigated Wnt-mediated mechanisms that control stemness by examining downstream target genes involved in the characteristic features of CSCs from initiation to metastasis.

## 2. Target Genes of Wnt/β-Catenin Signaling

**Figure 1:**
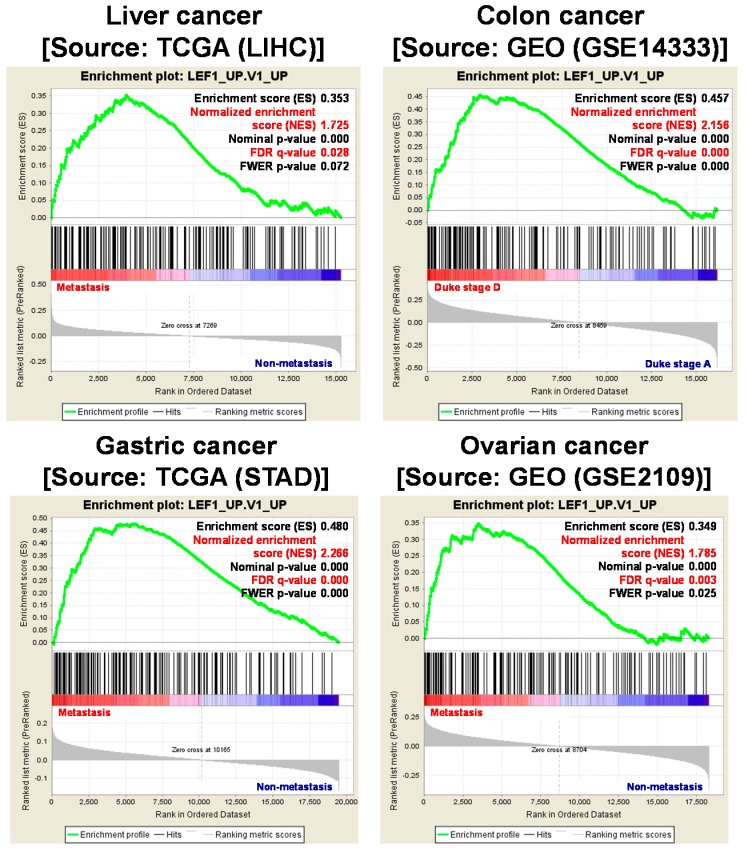
Enrichment of Wnt target genes in multiple metastatic cancer types. A group of genes that are induced by β-catenin/LEF1/TCF4 complex were identified through performed gene set enrichment analysis (GSEA). Microarray data of four different cancer, liver (LIHC), colon (GSE14333), gastric (STAD) and ovarian cancer (GSE2109), were analyzed. Genes upregulated by LEF1 were significantly enriched in metastatic cancers. The detailed methods are described in Supplementary Materials. TCGA; The Cancer Genome Atlas, LEF1; lymphoid enhancer-binding factor 1, GEO; Gene Expression Omnibus.

Wnt signaling regulates the expression of various genes through multiple pathways. In the canonical pathway, low β-catenin expression is maintained through phosphorylation of Ser/Thr residues and ubiquitination by protein degradation complexes. Following the stimulation of Wnt signaling by canonical ligands, degradation complexes are disrupted and de-phosphorylated β-catenin is translocated into the nucleus to bind LEF1/TCF4 family transcription factors, thereby transforming them from transcriptional repressors into transcriptional activators. In contrast, binding of non-canonical ligands to Wnt/FZD receptors transduces signals through intracellular calcium ions, c-Jun N-terminal kinases (JNK), receptor tyrosine kinase (RYK) or receptor tyrosine kinase-like orphan receptor (ROR), but not through β-catenin [15]. The canonical pathway has been studied more comprehensively than the non-canonical pathway and has been found to be activated by multiple mechanisms, including the destruction of degradation complexes, such as APC, and the consequent nuclear accumulation of β-catenin, in various types of cancer. Furthermore, the notion that CSCs possess higher activity of Wnt signaling covers canonical pathway as the LEF/TCF binding element showed higher transcriptional activity in CSCs than non-CSCs [16,17,18,19]. Therefore, to investigate the contributions of Wnt signaling to CSC regulation, we considered genes that are regulated by the canonical pathway and discuss their specific molecular mechanisms. Initially, we performed gene set enrichment analysis (GSEA) and identified a group of genes that are induced by β-catenin/LEF1/TCF4 complexes and are significantly upregulated in metastatic liver, colon, gastric and ovarian cancers compared with non-metastatic ones (Figure 1). In addition, we compared gene expression profiles in metastatic primary cancer (duke stage D) and early-stage non-metastatic primary cancers (duke stage A) using microarray data of colon cancers (GSE14333) and ovarian cancers (GSE2109) from the Gene Expression Omnibus. Subsequently, we identified genetic profiles that drive metastasis and applied gene expression data to GSEA to determine whether expression levels of the a priori defined set of genes differ significantly between the biological states. In these computations, genes that are upregulated by LEF1 were significantly enriched in metastatic cancers. Moreover, metastatic liver and gastric cancers showed increased expression of LEF1 target genes (TCGA). Thus, to further investigate the ensuing molecular mechanisms in CSC regulation, we generated target gene list from various source e.g., LEF1_UP.v1_UP gene set browsed from MSigDB, β-catenin target gene [20], and β-catenin chip assay result [21], and considered the biological functions of these genes and accordingly categorized them using Ingenuity Pathway Analysis (Ingenuity^®^ System, Available online: http://www.ingenuity.com, Redwood City, CA, USA). Subsequently, we interpreted these biological functions in terms of initiation, persistence, maintenance, resistance to anticancer therapy, invasion of neighboring tissues, and metastasis of CSCs (Table 1). The full list of gene (Table S1) and the detailed analytical method can be found in Supplementary Materials.

## 3. Initiation

Cancer initiation is associated with various intrinsic and extrinsic factors, including direct DNA damage by genotoxic compounds, viral infection, and inherent and acquired genetic mutations. Tumorigenesis is strongly associated with the presence of CSCs, which cause heterogeneity of cancer cells according to proposed hierarchical models [36]. Although the exact mechanisms behind the production of CSCs and acquisition of pluripotency remain unknown, transformation of normal stem cells into CSCs or re-acquisition of stemness in subpopulations of cancer cells have been related to Wnt signaling and the expression of its target molecules [37]. For example, the intestinal stem cell marker LGR5 is expressed in 5–10% of adenoma cells and is a direct Wnt target gene that is induced by direct β-catenin binding [21]. In addition, β-catenin accumulation upon APC loss increases RAC1 expression, leading to increased generation of reactive oxygen species (ROS) and nuclear transcription factor-κB (NF-κB) signaling, which are known to stimulate the expansion of LGR5^+^ cell populations [38]. The positive correlation between LGR5^+^ cells and Wnt activation was also confirmed in localization analyses of LGR5^+^ cells [13], which were transformed into neoplasia following nuclear accumulation of β-catenin due to APC deletion [13]. A recent study has also shown that LGR5 regulates the tumor-initiating ability of CSCs and that selective apoptosis of LGR5^+^ cells decreases tumor formation in human organoids [39]. Moreover, LGR5 is known to bind the Wnt receptor component R-spondin [37], creating a positive feedback loop between aberrant Wnt signaling and expansion of LGR5^+^ cell populations and contributing to tumor initiation [40].

DCLK1, the transcription factor, is regulated by Wnt signaling through the LEF-binding site at its promoter region [22] and has been shown to be enriched in metastatic liver and colon cancers [41,42,43]. DCLK1 is generally known as a marker for gastrointestinal tuft cells but has recently been considered a CSC marker because it is expressed in quiescent cancer cells and because high fractions of DCLK1^+^ cells exhibit the stem-like characteristics and the ability to overcome quiescence with sufficient niche signals [44,45]. Although DCLK1 has been associated with various signaling pathways, such as those mediated by Notch, Hedgehog, and NF-kB, the resulting control of stemness has not yet been clearly elucidated [46,47,48]. DCLK1 is positively correlated with nuclear translocation of β-catenin upon APC deletion [49], and β-catenin knockdown in APC^min/+^ mice leads to significant reduction in DCLK1 protein levels, which consequently attenuates crypt hyperplasia and tumorigenesis in APC^min/+^ mice without affecting downstream NF-kB signaling [50]. In addition, DCLK1 knockdown in APC^min/+^ mice attenuates intestinal adenoma and adenocarcinoma, whereas DCLK1 overexpression facilitates intestinal tumorigenesis in this model [51]. Wang et al. have investigated DCLK1 expression under normal and pathological conditions and have demonstrated their roles in colon cancer initiation [45]. In their study, DCLK1 expression was specifically increased in long-lived tuft cells, which originated from LGR5^+^ stem cells. Long-lived DCLK1^+^ tuft cells were also involved in regeneration upon chemical or microbial-induced injury and likely contributed to colon cancer as a tumor-initiating population with persistent Wnt activation [45]. Taken together, these studies have suggested that DCLK1 is a downstream molecule of the Wnt signaling pathway and is associated with direct LEF binding and cancer initiation.

Other CSC biomarkers include cluster of differentiation 44 (CD44), cluster of differentiation 133 (CD133), and aldehyde dehydrogenase (ALDH), which are positively regulated by Wnt/β-catenin signaling. Accordingly, compared with non-CSCs, ALDH^+^ or CD44^+^/CD24^−^ breast CSCs exhibit higher TCF/LEF-dependent transcriptional activity and treatment with Wnt3a further increases relative numbers of CSCs, whereas Wnt ligand knockdown decreases them [52]. Similarly, relative numbers of ALDH1^+^/CD133^+^ liver CSCs decrease following the blockade of β-catenin-dependent transcription [8]. However, further studies are needed to determine whether these CSC biomarkers are involved in Wnt-mediated generation of CSCs.

## 4. Persistence

Both CSCs and non-CSCs can grow persistently, whereas only CSCs adapt to the surrounding environment to avoid cell death. Accordingly, small subpopulations of CSCs of patients have been maintained for years in differentiated culture systems, and non-CSCs can be de-differentiated to CSCs using cytokine supplements under appropriate culture conditions [53,54] and non-CSCs are reportedly derived from persistent CSCs [55] which proliferate more rapidly, express anti-apoptotic genes, and have consequent higher survival rates. In a recent study, genetic disruption of proliferation was found to deplete the maintenance of CSC populations remaining non-CSCs population intact among human epithelial breast and colon cancer cells [56]. CSCs have also been shown to resist the effects of radio- and chemotherapy conditions, which are extremely deleterious for tumor and surrounding cell populations [5].

Kruppel-like factor (KLF5) is a zinc-finger transcription factor that is critical for maintaining stem cell integrity and regulating the cell cycle [57]. The KLF5 gene is upregulated in various cancer types, including hepatocellular carcinoma and breast and intestinal cancers, and KLF5 deletion in the presence of induced mutant β-catenin suppresses the transcription of histone deacetylase 1 (HDAC1), which promotes colorectal cancer by promoting both stem and transit-amplifying cell proliferation [57]. KLF5 is constantly upregulated in CD44^high^/CD133^high^ CSC populations, and KLF5 overexpression enhances colony-forming ability and resistance to anticancer drugs, such as cisplatin and 5-fluorouracil, reflecting increased anchorage-independent growth [58]. Regulatory roles of KLF5 on cancer stemness have also been indicated in siRNA-mediated KLF5-knockdown experiments that have shown reduced numbers of CD44^high^/CD133^high^ cells [58]. In mice, inducible deletion of KLF5 in LGR5^+^ stem cells suppresses their proliferation and survival in association with nuclear localization of β-catenin and the generation of abnormal apoptotic cells in intestinal crypts [57].

Endothelin1 (EDN1) is primarily expressed in vascular epithelial cells, where it maintains vascular tone. EDN1 is reportedly secreted by many solid tumors, and it induces persistent growth and survival by promoting cell proliferation and suppressing apoptosis. Although the mechanisms that lead to pathological EDN1 levels are unclear, β-catenin suppression has shown to be closely associated with reduced EDN1 mRNA expression [23]. Moreover, END1 peptide secretions from colorectal cancer cells are significantly reduced after the introduction of dominant-negative TCF4 or β-catenin mutants, whereas overexpression of wild-type β-catenin or TCF results in increased END1 peptide production. In addition, treatments of APC-induced growth arrested cells with exogenous EDN1 restore proliferation by suppressing cell death. Furthermore, END1 overexpression enhances cell proliferation in vitro and in xenotransplantation assays and is accompanied with increases in the expression of several cell cycle and proliferation molecules [59]. Chip assays and sequencing analyses have also indicated direct regulation of EDN1 by β-catenin, and four potential TCF-binding elements have been identified in the EDN1 promoter region. Subsequent luciferase reporter assays have confirmed that β-catenin directly regulates EDN1 expression through these TCF-binding elements [23], and Rosano et al. have shown that EDN1 and its receptor are upregulated in cisplatinum-resistant and taxo-resistant cell lines, reflecting decreased sensitivity to cytotoxic drugs and increased survival and proliferation due to enhanced MAPK and Akt signaling [60].

Achaete-scute homolog 2 (ASCL2) is an intestinal stem cells transcription factor that contains a basic helix-loop-helix domain and a downstream Wnt signaling target [61]. ASCL2 overexpression is observed in colorectal cancers [62], and ASCL2 overexpression shifts the hierarchy of stem/progenitor cells in liver metastases and affects clinical outcomes [63]. Hence, dysregulated ASCL2 expression is thought to facilitate colorectal cancer cell proliferation [64,65]. Elevated ASCL2 expression in patients with osteosarcoma is also associated with osteosarcoma metastasis and poor prognosis [66]. Hence, with β-catenin and TCF, ASCL2 activates genes that are fundamental for maintaining the stem cell state, suggesting that ASCL2 forms a transcriptional switch that is Wnt-responsive and Wnt-dependent and defines stem cell identity. Moreover, ASCL2 is regulated in a direct autocrine loop that leads to distinct on/off expression patterns, and Wnt/R-spondin reportedly activates this regulatory loop [24]. Lastly, compared with control cells, ASCL2-overexpressing cells exhibit 5-FU resistance due to greater overall survival and fewer apoptotic cells [67].

As a member of the activator protein-1 superfamily, Fos-related antigen-1 (FRA1) positively regulates transcription and post-transcriptional processes. Accordingly, elevated FRA1 mRNA levels are associated with aberrant β-catenin accumulation in lung [68], brain [69], breast [70], and bladder [71] cancers. Furthermore, β-catenin accumulation and FRA-1 are positively correlated with WHO disease grades [69], and Chip assays have shown direct biding of β-catenin and FRA1 [21]. In experimental systems, FRA1 overexpression is sufficient to increase cell motility and anchorage-independent growth, and these are representative features of stem cells. In addition, compared with FRA1 expression, FRA1 knockdown prevents cell cycle progression, cell growth, and colony formation under anchorage-independent conditions and increases sensitivity to cisplatin [72]. At the protein level, FRA1 overexpression reduces p53 and increases MDM2 expression levels, consistent with apoptosis inhibition [68].

The transcription factor Myc proto-oncogene protein (MYC) favors cell growth and proliferation and suppresses cell cycle arrest [73,74]. These roles of MYC have been well documented in lymphocytes using conditional knockout systems [75,76]. In addition, MYC overexpression in B lymphocytes is sufficient to increase cell proliferation [77], and ectopic expression of MYC in hepatocytes using an adenoviral gene transfer system in vivo results in significant cell growth [78]. MYC is reportedly essential for Wnt-mediated growth, and its absence reduces Wnt-induced cell growth and proliferation in colonic crypts [78]. MYC can also switch metabolic phenotypes between aerobic glycolysis and oxidative phosphorylation by directly upregulating the transcription of glucose transporter-1, hexokinase-2, enolase, and lactate dehydrogenase [79]. In general, highly proliferative cancer cells depend on aerobic glycolysis rather than oxidative phosphorylation, and the ensuing rapid energy and lactic acid production is known as the Warburg effect [80]. Compared with non-CSCs, CSCs prefer Warburg-type energy metabolism, thereby allowing constitutive proliferation and resistance to cell damage by radiation-induced ROS [80,81]; this potentially reflects scavenging of ROS by abundant lactate in these cells [82]. In addition to metabolic and proliferative contributions to stemness, CSCs have to escape immune surveillance. MYC has been shown to directly regulate the expression of the tumor cell surface immune checkpoint protein cluster of differentiation 47 (CD47) and programmed death-ligand 1 (PD-L1), which mediate “don’t eat me” and “don’t find me” signals, respectively, following MYC binding to their promoters. MYC inactivation in mouse tumors also leads to decreased CD47 and PD-L1 expression and enhanced antitumor immune responses. However, MYC inactivation in tumors due to enforced CD47 or PD-L1 expression leads to suppressed immune responses and continued tumor growth [83,84]. Thus, MYC likely initiates and maintains tumorigenesis, in part, by modulating immune regulatory molecules.

Cyclin D1 (CCND1) is a traditional Wnt/β-catenin target gene, and time-lapse photography and quantitative image analyses have shown that CCND1 facilitates continued cell cycle progression [85]. This study has also revealed a highly ordered mechanism underlying continued proliferation, in which the decision to continue the cell cycle occurs at the G2 phase under conditions of elevated CCND1 expression. These conditions are maintained through the G1 phase and are required for initiation of the S phase, during which CCND1 levels are automatically reduced to allow DNA synthesis. High CCND1 expression is again required upon entry into the G2 phase. Consistent with this fact, CCND1 expression was found to be higher in metastatic lymph nodes than in primary regions of papillary thyroid carcinomas that were co-localized with β-catenin [86]. Another study demonstrated that CCND1 expression is upregulated in lung CSCs and promotes cell proliferation and clonogenic formation [87] in association with resistance against progestin [88] and cisplatin [89]. Collectively, these studies have indicated that Wnt-mediated re-colonization at distant orgnas is partly dependent on CCND1.

Chemo-resistance has also been associated with the ATP-binding cassette subfamily B member 1 (ABCB1), which is an ATP-dependent drug efflux pump that transports molecules across cell membranes. ABCB1 overexpression and its product P-glycoprotein are responsible for multiple drug resistance (MDR). The basal promoter of ABCB1 has several β-catenin/TCF4/LEF1-binding sites, suggesting that the canonical Wnt/β-catenin pathway regulates ABCB1, as shown in early colorectal [25], neuroblastoma [90] and breast [91] cancers. Furthermore, ABCB1 is highly overexpressed in doxorubicin-resistant cell lines [90], and β-catenin depletion in a chronic myeloid leukemia cell line leads to reduced ABCB1 mRNA expression, whereas Wnt signaling increases ABCB1 mRNA expression [92]. Taken together, these studies have indicated that resistance in CSCs can be achieved indirectly by mechanisms that lead to constitutive proliferation and resistance to apoptosis or directly by defense mechanisms that desensitize CSCs to anticancer agents and facilitate persistent growth.

## 5. Invasion and Migration

Although the origin of CSCs remains controversial, re-acquisition of CSC phenotypes in differentiated non-CSCs has been reported in multiple studies, and EMT is considered a major driver. EMT is a crucial process for morphogenesis during embryonic development and was initially recognized by developmental biologists. However, studies conducted in the last decade have shown that EMT can be aberrantly rebooted in adult tissues under pathological conditions, such as fibrosis and cancer in terms of poor wound healing and cancer invasiveness, respectively. EMT also contributes to the acquisition of invasion potential during breast carcinogenesis and chemotherapy. Specifically, during EMT, morphologic and phenotypic changes increase cancer cell motility, dissemination, invasiveness, and dedifferentiation [93,94]. Multiple signaling pathways are involved in the EMT process, and their downstream transcription factors, such as Snail, Twist, and Zeb, function as master controllers of the EMT process [95]. Moreover, among various signaling pathways, Wnt/β-catenin signaling is known to strongly promote EMT through LEF/TCF-dependent transcriptional activation [96]. Wnt/β-catenin activation is also required for generating CSCs from de-differentiated non-CSCs. In particular, inhibition of glycogen synthase kinase-3β (GSK-3β) expression promotes nuclear translocation of β-catenin and restores stem cell phenotypes in differentiated colorectal cells [97]. Moreover, activation of the E-twenty-six-related gene–FZD4 axis causes nuclear accumulation of β-catenin and transforms prostate cancer cells through EMT [98]. More recently, it has been confirmed that β-catenin forms a complex with Twist1/TCF4 and enhances its transactional activity, and consequently promotes tumorigenesis and re-acquisition of the CSC phenotype [99]. The invasion potential of CSCs is reportedly higher than that of non-CSCs, and this has been demonstrated in established cancer cell lines and patient-derived cancer cells. These experiments have indicated that glioma CSCs exhibit higher invasion activity than non-CSCs in the frontal region of tumors [100]. Similarly, ECM matrix penetration and cellular motility are enhanced in various types of CSCs, including those from breast [101] and liver [102] cancers, and some Wnt/β-catenin target genes have been shown to promote CSC invasion and migration.

Matrix metalloproteinase-7 (MMP7) is a well-known β-catenin target gene. Accordingly, ectopic LEF1 expression increases β-catenin binding to TCF-binding sequences and activates the MMP7 promoter [26]. Moreover, the conserved LEF1 recognition site is present on the promoter region of MMP-7 and is bound directly by LEF1 in oral squamous cell carcinoma cell lines and human oral squamous cell carcinoma tissues [103]. Members of the MMP family contribute to extracellular matrix (ECM) degradation and are classified according to their substrates as gelatinases (MMP2 and MMP9), collagenases (MMP1 and MMP13), metalloelastase (MMP12), and matrilysin (MMP7) [104]. MMP7 is synthesized and secreted into the extracellular matrix to increase invasion potential of cells via proteolytic degradation of ECM proteins. Although most MMPs degrade their ECM substrates, MMP7 is expressed in many epithelial cell types [105] and is reportedly expressed at high levels in multiple cancer types, including pancreatic [106], colon [107], and gastric [108] cancers. In particular, MMP7 is associated with highly invasive phenotypes that lead to poor prognoses in cancer patients. In addition to ECM degradation, MMP7 promotes cancer invasion by converting inactive forms of other MMPs, such as MMP2 and MMP9, into active forms [104]. MMP7 is involved in Wnt-mediated invasion in cancer cells. Accordingly, Wnt signaling activation by transfection with a mutant β-catenin that lacks the GSK-3β-specific phosphorylation site leads to morphological changes in oral squamous cancer cells from polygonal shape to spindle shape and decreases cell–cell interactions of cells with high invasion and migration capacity by inducing MMP7 [103]. These studies have implicated MMP7 as a mediator of increased CSC invasion potential in response to Wnt/β-catenin signaling. Similarly, membrane type 1-matrix metalloproteinase 1 (MT1-MMP) is directly regulated by the β-catenin/TCF complex through direct binding to its TCF promoter-binding site [109]. MT1-MMP increases invasion capacity through actin-rich cell protrusions that are known as invadopodia and are responsible for matrix degradation [110]; ectopic MT1-MMP overexpression promotes EMT [111]. Furthermore, CD133^+^ ovarian CSCs exhibit high MT1-MMP expression, and MT1-MMP knockdown specifically inhibits CSC invasiveness without affecting non-CSCs [112]. These experiments have indicated that MT1-MMP is a target of Wnt/β-catenin signaling that facilitates CSC infiltration into surrounding tissues.

In a previous study, inhibition of hyaluronan (HA) synthase-2 (HAS2), a biosynthetic enzyme of hyaluoran which is known as one of MMP7 regulators, was shown to decrease MMP7 expression under the condition of HAS2 inhibition and consequently inhibit the invasion ability of colon cancer cells [113]. HAS2 expression is also higher in bone-metastatic sub-clone breast cancer cells than in parental cells, and it increases the perforation of basement membranes by enhancing MMP activities without affecting their expression, reflecting inhibited expression of tissue metalloproteinase inhibitor 1 [114]. HAS2 expression is regulated by Wnt/β-catenin signaling, and LEF1 overexpression increases HAS2 mRNA expression in colon cancer cell lines by through its multiple binding sites on the promoter region of HAS2. Furthermore, HAS2 protein levels are indirectly increased by LEF1 via enhanced expression of HAS2 antisense RNA, which stabilizes HAS2 mRNA [27]. Consistent with this finding, breast CSCs with invasive phenotypes have been shown to express HAS2 at high levels, and subsequent treatment with the HAS2 inhibitor 4-methylumbelliferone has been shown to decrease the ability of CSC migration and invasion [115].

HA biosynthesis is activated as a consequence of increased HAS2 expression following Wnt signaling activation. Thereupon, the extracellular matrix components enter into HA-rich condition. As CD44 has HA binding site in its extracellular domain, HA binds to CD44 and transfers signals that mediate cellular responses to microenvironments, thereby increasing its motility [116]. This HA-induced invasive phenotype is dependent on C-X-C chemokine receptor type 4 (CXCR4) at least in part, due to its nearby location of HA binding site on CD44. The binding of HA on CD44 facilitates CXCR4 activation with its ligand, C-X-C motif chemokine ligand 12 (CXCL12), which consequently enhances MMP expression and CSC invasion [117]. Singh et al. revealed that the induction of CXCL12 enhanced the expression of MMP family members such as MMP1, MMP13, MMP9, MMP3, MMP10, MMP11 and MMP14 which are able to destruct the extraceullar matrix to increase invasion potentials [117]. For several decades, CD44 has been considered as one of the traditional Wnt target gene because CD44^+^ populations tend to expand when Wnt signaling is activated by APC mutation. In contrast, CD44^+^ populations are depleted by abrogation of the TCF4/β-catenin complex following genetic disruption of the TCF4/β-catenin-binding site [28]. Similarly, under HA-rich conditions, CD44^+^ populations frequently bound to CXCR4/CXCL12 [118], suggesting that indirect CXCR4 regulation by Wnt and HAS2 may increase the invasiveness of CD44^+^ CSCs with TCF/β-catenin-dependent expression.

Moreover, the direct regulation of Wnt on CXCL12 has been confirmed in previous studies [29]. Specifically, under conditions of Wnt activation in stem-like basal cancer cells, β-catenin binds to the promoter region of CXCL12 via a LEF/TCF-binding site and increases its mRNA expression. Moreover, this signaling mechanism is inhibited following β-catenin disruption by treatment with ICG-001 [29]. In another study, dense intra-tumoral microvessels have been observed near CD133^+^ glioma CSCs that co-express the CXCR4 [119], and one study has shown that CXCR4^+^ subpopulations of CD133^+^ pancreatic CSCs can evade primary tumors and disseminate into the blood stream [120]. Various experiments using CXCR4 inhibitors have suggested that CSCs exploit the CXCR4/CXCL12 axis to induce the secretion of vascular endothelial growth factor and further promote invasion into vessels [121,122]. Collectively, these studies have indicated that Wnt/β-catenin in CSCs promote invasive phenotypes via CXCR4/CXCL12 axis-mediated angiogenesis and lymphangiogenesis. Subsequently, invasive CSCs penetrate into surrounding tissues and into proximal blood and lymphatic vessels, leading to systemic distribution in the circulation system and arrival at metastatic sites.

## 6. Metastasis

Both cancer cell and CSC populations are heterogeneous, and CSC subpopulations with higher metastatic potency are likely to be disseminated into nearby tissues. In addition, migration to distant sites through the blood stream leads to the colonization of secondary organs and outgrowth. Circulating disseminated CD44^+^/CD24^−/low^ or ALDH1^high^/CD24^−/low^ expressing CSCs have been discovered in patients with metastatic breast cancer [123]. Similarly, disseminated CD133-expressing CSCs have been identified in patients with metastatic prostate [124] and recurrent colon [125] cancer. During circulation in the blood stream or at pre-metastatic niches, CSCs maintain dormancy and increase survival potential. Although the regulatory roles of Wnt in dormancy remain controversial, a recent study has suggested that Wnt-related mechanisms are involved in the regulation of latent competency, which is a dormant state with metastatic potential. Specifically, following Wnt signaling activation, the CXCR4/CXCL12 axis contributes to survival and dormancy status. In addition, analyses of patients with breast cancer have indicated that the expression of proto-oncogene tyrosine-protein kinase (Src) is positively correlated with late relapse in bone, but not in other tissues, and further mechanistic studies have shown that CXCR4/CXCL12-activated Src supports the survival of indolent breast cancer cells in bone marrow by activating Akt [126].

Dickkopf-related protein (DKK1) is a Wnt downstream molecule and a negative feedback component that likely plays essential roles in the regulation of late competency. Upon activation, Wnt reportedly binds TCF in the promoter region of DKK1 and induces its expression [31]. Li et al. have shown that instead of directly binding to the Wnt ligand, DKK1 antagonizes Wnt signaling by forming a complex with LDL receptor related protein 6 (LRP6) and Kremen and then removing LRP6 from the cell surface, thereby promoting its degradation and inhibiting Wnt binding and LRP6 turnover [127]. As an inhibitor of this Wnt autocrine loop, DKK1 decelerates the cell cycle and reduces the expression of innate immune sensors, allowing evasion of NK-cell-mediated immune surveillance [128]. Taken together, these data have indicated that Wnt activation may be essential for CSC transformation into dormancy and that it likely acts by upregulating DKK1 expression.

Moreover, one study has indicated that Wnt/β-catenin signaling controls metastatic colonization of target organs and that human lung adenocarcinomas enhance the competence of lung adenocarcinoma cells to colonize bone and brain tissues using distinct Wnt signaling pathways through LEF1 and HOXB9 [129]. During re-colonization at distant organs, CSCs escape from dormancy and re-initiate proliferation. This can be achieved by direct transcriptional activation in CSCs or by stimulation from cells, such as fibroblasts and immune cells, in the microenvironment. Wnt/β-catenin signaling can re-initiate cell cycle progression in dormant CSCs by upregulating c-MYC (MYC) expression through direct promoter binding [130,21]. Accordingly, MYC is central to the core interactome in metastatic breast cancer patients, and stable MYC knockdown decreases colony formation at secondary organs [131]. These studies have suggested that MYC mediates Wnt-induced metastatic colonization by re-initiating the cell cycle and cell growth.

Claudin1 (CLDN1) is a tight junction protein (TJP) that regulates the permeability of epithelial barriers through which small ions and neutral solutes enter [132]. Recently, the roles of CLDN1 in metastasis have received increasing attention because increased permeability of endothelial cells is required for cancer cells to intravasate and extravasate [133]. Moreover, CLDN1 expression in cancer cells is positively correlated with cancer progression because TJP is essential for cell–cell interactions that promote carcinogenesis and metastasis [134]. However, although CLDN1 overexpression in lung cancer cells increases cell–cell connections and prevents dissemination into proximal tissues [135], CLDN1 induces EMT in hepatocellular carcinomas and promotes invasion and metastasis by activating cellular signaling via Ras and extracellular signal-regulated kinases [136]. Moreover, inhibition of CLDN1 expression in gastric cancer cells results in decreased re-initiation of proliferation and leads to anoikis [137]. Similarly, CLDN1 augments anchorage-independent growth to favor metastatic colonization. However, clinical evidence has indicated that CLDN1 is a poor prognostic marker because higher CLDN1 levels are present in tumor tissues from distant metastatic sites in patients than in matched primary tumors [138,139]. Although further studies are needed to elucidate the precise mechanisms of CLDN1 in CSCs, resistance to anoikis is a typical phenotype of CSCs and is essential for anchorage-independent survival and colonization. Accordingly, CLDN1 expression is increased in CSCs compared with that in non-CSCs [140], and CLDN1 is directly upregulated by Wnt signaling. Specifically, ectopic APC expression decreases CLDN1 expression in Wnt-activated APC-deficient colon cancer cells, and CLDN1 requires its TCF-binding site for transcriptional activation [32]. In addition, the typical intestinal transcription factor caudal homeobox protein binds to the promoter of CLDN1 and increases its expression by forming a complex with TCF4 [141]. Hence, CLDN1 may mediate Wnt-induced re-initiation of CSCs at metastatic sites. Collectively, these studies have indicated that Wnt signaling re-activation at secondary organs can induce CLDN1 overexpression and trigger dormant CSC proliferation by promoting resistance to anoikis.

Metastatic CSCs has recently emerged which refers the specific subpopulations of CSCs that colonize better at the distant organs than other cell populations [142,143]. Therefore, various ongoing studies are directed at the discovery of biomarkers for metastatic CSCs, and the variant isoforms of CD44 (CD44v) have gained attention as one of novel biomarkers. CD44v are generated through alternative splicing from CD44 and gain the additional extracellular domains providing more glycosylation sites which enhance the ability of capturing various microenvironmental ligands including hepatocellular growth factor (HGF), osteopontin (OPN), vascular endothelial growth factor (VEGF), fibroblast growth factors (FGF), and CXCL12. Hence, these ligands are concentrated nearby CD44v and activate their downstream signalings such as PI3K-Akt, Smad, Src, and β-catenin pathway promoting metastasis in CD44v^+^ CSC [116,118,144]. For these reasons, CD44v makes CSCs respond to microenvironmental cytokines facilitating metastatic signalings via diverse pathways. Among existing numerous variants, CD44v6^+^ CSCs are recently documented as a subpopulation that forming outgrowths at distant metastatic sites by exhibiting constitutive reprogramming [33]. The specific inhibition of CD44v6 using antibodies have proved that metastasis of head and neck cancer is dependent of CD44v6^+^ CSCs [118]. Interestingly, in colon cancer, CD44v6^+^ CSCs are more frequently observed from the secondary organ outgrowth site with elevated β-catenin accumulation level, while CD44v6^+^ CSCs are hardly observed from the primary tumor site [33]. In addition, the presence of CD44v6^+^ populations was significantly decreased under the conditions of TCF4 loss [28]. Accordingly, the genetic profiles present that Wnt-signaling molecules are up-regulated in CD44v6^+^ CSCs compared to that of CD44v6^−^ counterpart, and the LEF/TCF-dependent transcriptional activity is activated in CD44v6^+^ CSCs as well [33]. Moreover, Wnt3a ligand-mediated activation of Wnt signaling increases the existing population of CD44v6^+^ CSCs [33]. From these together, Wnt signaling enhances metastatic potential of CSCs through CD44v6 which activates metastatic signaling exhibiting more sensitive response to microenvironment.

In pre-metastatic niches, various components of secondary microenvironments can stimulate CSC colonization by activating Wnt signaling. For example, the extracellular matrix protein tenascin C is commonly found in stem cell niches and supports Wnt-mediated outgrowth of breast cancer micrometastases by increasing the expression of Syndecan 4, which has been implicated as a co-receptor of the Wnt receptor FZD7 [145,146,147]. Periostin is another matrix protein in stem cell niches that promotes the outgrowth of micrometastatic colonies by facilitating Wnt ligand secretion from tumor cells [148]. Tumor-associated macrophages also secrete interleukin-1β, which activates Wnt signaling in colon cancers by phosphorylating GSK-3β, stabilizing β-catenin, and enhancing TCF target gene expression [149]. Conversely, Wnt signaling contributes to microenvironments that favor re-colonization by CSCs. Wnt 7a is a canonical ligand of the Wnt pathway [150,151] and is required for the recruitment of cancer-associated fibroblasts that enhance invasion and metastatic potential of cancer cells [152]. Interestingly, the common ECM protein fibronectin (FN1) is directly upregulated by Wnt signaling through LEF/TCF-binding sites on its promoter region [34]. Moreover, FN1 promotes organ-specific metastasis in which hematopoietic progenitor cells expressing the FN1 receptor integrin α4β1 migrate and adhere to FN1-rich regions through ligand–receptor bonds and subsequently produce MMP-9 [153].

Lastly, Wnt signaling activation facilitates immune surveillance evasion through Cyclooxygenase-2 (COX2), which is directly upregulated by Wnt through its LEF/TCF promoter-binding site [35] and produces prostaglandin E2 (PGE2) to convert CD4^+^ T cells to regulatory T cells, thereby inducing apoptosis of CD8^+^ cytotoxic T cells [154]. Through this molecular mechanism, Wnt activation can promote bone metastasis of breast cancers by aggregating dead CD8^+^ cytotoxic T cells [30]. Collectively, current data indicate that Wnt signaling is a potent inducer of metastasis, induces CSC dormancy during circulation in the blood, re-initiates outgrowth of CSCs at secondary organs, and modulates microenvironments to favor CSCs.

## 7. Conclusions

Wnt signaling is considered as a major contributor to CSC biology. In this review, we summarized and characterized Wnt signaling mechanisms that regulate CSCs from initiation to metastasis. Initially, continuously upregulated Wnt signaling converts normal stem cells and differentiated cells into CSCs. Subsequently, abnormally hyperactivated Wnt signaling allows entry into the early stages of metastasis and then facilitates persistent growth, invasion, migration, and homing. Negative feedback of Wnt signaling pathway then induces CSC dormancy, and subsequent hyperactivated Wnt signaling is central to re-initiation and colonization of metastatic sites. Although Wnt signaling inhibitors have been developed in multiple studies, their use is limited by the involvement of Wnt signaling in homeostasis and development, leading to potential side effects. Moreover, Wnt signaling is dynamic throughout the process from initiation to metastasis, complicating the timing of therapeutic interventions that target Wnt. Therefore, further comprehensive studies on the downstream mechanisms of Wnt are required to develop novel therapeutic agents.

## Figures and Tables

**Table 1:** Summary of Wnt target genes and their biological functions in cancer stem cells (CSCs).

Biological Function	Gene	Full Name	Direct/Indirect Target	Reference
Initiation	*LGR5*	Leucine-rich repeat-containing G-protein-coupled receptor 5	Direct	[21]
	*DCLK1*	Doublecortin-like kinase	Direct	[22]
Persistence	*KLF5*	Krueppel-like factor 5	Direct	[21]
	*EDN1*	Endothelin-1	Direct	[23]
	*ASCL2*	Achaete-scute homolog 2	Direct	[24]
	*FRA1*	Fos-related antigen 1	Direct	[21]
	*MYC*	Myc proto-oncogene protein	Direct	[21]
	*CCND1*	CyclinD1	Direct	[21]
	*ABCB1*	ABC multidrug transporter	Direct	[25]
Invasion	*MMP7*	Matrix Metallopeptidase 7	Direct	[26]
	*HAS2*	Hyaluronan synthase-2	Direct	[27]
	*CD44*	Cluster of differentiation 44	Indirect	[28]
	*CXCL12*	C-X-C motif chemokine ligand 12	Direct	[29]
	*CXCR4*	Chemokine receptor type 4	Direct	[30]
Metastasis	*CXCL12*	C-X-C motif chemokine ligand 12	Direct	[29]
	*CXCR4*	Chemokine receptor type 4	Direct	[30]
	*DKK1*	Dickkopf-related protein 1	Direct	[31]
	*CLDN1*	Claudin-1	Direct	[32]
	*CD44v6*	Cluster of differentiation 44 variant exon 6	Indirect	[33]
	*FN1*	Fibronectin	Direct	[34]
	*COX2*	Cyclooxygenase-2	Direct	[35]

## Supplementary Materials

Supplementary materials can be found at www.mdpi.com/1422-0067/18/8/1604/s1.

## Abbreviations

LEF1	Lymphoid Enhancer-binding factor 1
CSC	Cancer stem cell
FZD	Frizzled receptor
TCF	T-cell factor
APC	Adenomatous polyposis coli
LGR5	Leucine-rich repeat-containing G-protein-coupled receptor 5
DCLK1	Doublecortin-like kinase 1
EMT	Epithelial-to-mesenchymal transition
JNK	c-Jun N-terminal kinases
RYK	Receptor tyrosine kinase
ROR	Receptor tyrosine kinase-like orphan receptor
GSEA	Gene set enrichment analysis
ROS	Reactive oxygen species
NF-κB	Nuclear transcription factor-κB
CD44	Cluster of differentiation 44
CD133	Cluster of differentiation 133
ALDH	Aldehyde dehydrogenase
KLF5	Kruppel-like factor
HDAC1	Histone deacetylase 1
EDN1	Endothelin 1
ASCL2	Achaete-scute homolog 2
FRA1	Fos-related antigen-1
CD47	Cluster of differentiation 47
PD-L1	Programmed death-ligand 1
CCND1	Cyclin D1
ABCB1	ATP-binding cassette subfamily B member 1l
MDR	Multiple drug resistance
GSK-3β	Glycogen synthase kinase-3β
MMP7	Matrix metalloproteinase-7
ECM	Extracellular matrix
MT1-MMP	Membrane type 1-matrix metalloproteinase 1
HA	Hyaluronan
HAS-2	Hyaluronan synthase-2
CXCR4	C-X-C chemokine receptor 4
CXCL12	C-X-C motif chemokine ligand 12
Src	Proto-oncogene tyrosine-protein kinase
DKK1	Dickkopf-related protein
LRP6	LDL receptor related protein 6
CLDN1	Claudin1
TJP	Tight junction protein
CD44v	CD44 variant
HGF	Hepatocellular growth factor
OPN	Ostepontin
VEGF	Vascular endothelial growth factor
FGF	Fibroblast growth factors
FN1	Fibronectin
COX2	Cyclooxygenase 2
PGE2	Prostaglandin E2
